# Fall prevention: is the STRATIFY tool the right instrument in Italian Hospital inpatient? A retrospective observational study

**DOI:** 10.1186/s12913-017-2583-7

**Published:** 2017-09-15

**Authors:** Greta Castellini, Antonia Demarchi, Monica Lanzoni, Silvana Castaldi

**Affiliations:** 1 Department of Biomedical Sciences for Health, University of Milan, Via Pascal, 36, 20133 Milan, Italy; 2 Unit of Clinical Epidemiology, IRCCS Istitute Orthopedic Galeazzi, Milan, Italy; 3 Fondazione IRCCS Ca’ Granda Ospedale Maggiore Policlinico, Milan, Italy

**Keywords:** Accidental falls, Incident reporting, Patient safety, Risk assessment tools

## Abstract

**Background:**

Although several risk assessment tools are in use, uncertainties on their accuracy in detecting fall risk already exist. Choosing the most accurate tool for hospital inpatient is still a challenge for the organizations. We aimed to retrospectively assess the appropriateness of a fall risk prevention program with the STRATIFY assessment tool in detecting acute-care inpatient fall risk.

**Methods:**

Number of falls and near falls, occurred from January 2014 to March 2015, was collected through the incident reporting web-system implemented in the hospital’s intranet. We reported whether the fall risk was assessed with the STRATIFY assessment tool and, if so, which was the judgement. Primary outcome was the proportion of inpatients identified as high risk of fall among inpatients who fell (True Positive Rate), and the proportion of inpatients identified as low-risk that experienced a fall howsoever (False Negative Rate). Characteristics of population and fall events were described among subgroups of low risk and high risk inpatients.

**Results:**

We collected 365 incident reports from 40 hospital units, 349 (95.6%) were real falls and 16 (4.4%) were near falls. The fall risk assessment score at patient’s admission had been reported in 289 (79%) of the overall incident reports. Thus, 74 (20.3%) fallers were actually not assessed with the STRATIFY, even though the majority of them presented risk recommended to be assessed. The True Positive Rate was 35.6% (*n* = 101, 95% CI 30% - 41.1%). The False Negative Rate was 64.4% (*n* = 183, 95% CI 58.9%–70%) of fallers, nevertheless they incurred in a fall. The STRATIFY mean score was 1.3 ± 1.4; the median was 1 (IQQ 0–2).

**Conclusions:**

The prevention program using only the STRATIFY tool was found to be not adequate to screen our inpatients population. The incorrect identification of patients’ needs leads to allocate resources to erroneous priorities and to untargeted interventions, decreasing healthcare performance and quality.

## Background

Falls and falls related injuries are a significant public health issue. A fall is defined as “an event which results in a person coming to rest inadvertently on the ground or floor or other lower level”, [1]. The accidental fall is the most common adverse event among hospital inpatients and it occurs from 0.3–19 per 1000 patient-days, [2, 3]. The National Institute for Health and Care Excellence (NICE) has reported nearly 209.000 falls between 1 October 2011 and 30 September 2012 in England with a relative cost of £2.3 billion a year, [4]. The fall burden is remarkable: injuries, fractures, anxiety, depression are some of the individual and social fall consequences, [5]. Death, for instance, is the most common unintentionally injury due to a fall in people over 65 years, [6]. Due to significant physical, psychological and socio-economic costs, fall prevention has been recognized as a fundamental process for health care interventions. The Joint Commission International has defined the reduction of patient harm for falls as an indicator of care quality in a hospital and a National Patient Safety Goal Standard, [7]. One of the common strategies in the fall reduction process is to use a fall risk assessment tool to identify the population at risk of fall, [8]. Therefore, it seems important to assess risk of falling using ad hoc tools and to adopt the perfect strategy to implement them into the organizational routine. The choice of the most adequate and adaptable tool for hospital inpatient population is always a challenge for the organizations. Several studies have assessed the accuracy of the existing risk assessment tools however, strong evidence does not still exist and doubts on their usefulness are real, [3, 9].

A frequently applied tool in hospitals prevention programs, mainly with old inpatients, is the St. Thomas Risk Assessment Tool in Falling elderly inpatients (STRATIFY), [10]. This tool comprises five items addressing risk factors: past history of falling, patient agitation, visual impairment, incontinence, transfer and mobility, [11]. The STRATIFY score range from 0 to 5 points and the predictive cut off of risk of falling is a score ≥ 2 points. Oliver et al. [11] identified that sensibility and specificity were, respectively, 92.4% and 68.3% in the acute and rehabilitation settings, whereas some authors declared that the STRATIFY’s predictive proprieties fit best medical inpatients younger than 65 years old and it’s unsuccessful in more elderly inpatients, [10]. This tool has been object of studies performed in several settings with deluding findings about its usefulness in screening faller than non-fallers, [3, 12]. For instance, the STRATIFY has been considered as not the best tool for screening in traumatic brain injury rehabilitation as well its accuracy has been doubted in the acute geriatric inpatient population, [13, 14]. Due to lack of demonstrable efficacy, limitations on the applicability of the instrument exist, [12, 15]. Nevertheless, the STRATIFY is suggested to be use in several hospitals as, for example, a regional guideline based on Italian Health Ministry’ recommendation proposed [16, 17].

Using an inaccurate tool may develop an untrue sense of safety in both patients and healthcare personnel. It can leave patient exposed to fall risk and to the potential adverse effects of falling.

A recent observational study compared the effectiveness of two assessment tools (Conley Scale and Hendrich Risk Model) in an Italian hospital but the evaluation was limited only to three inpatients populations, [9]. We aimed to retrospectively assess whether the STRATIFY risk assessment tool, implemented in a research and teaching hospital in North Italy, was appropriately able to detect inpatients fallers as at high risk and, consequently, to report all general characteristics, intrinsic and extrinsic factors related to the sample of fallers obtained. To our knowledge, this study is the first in the Italian health care system that retrospectively evaluates whether a preventive program with a known fall risk assessment tool adequately detects all hospital inpatient population who fell. The magnitude of the problem of falls is recognized by researchers and the public health community as a growing epidemic [18]. As a consequence, focusing on how risk of falling can be detected, limited and managed in hospitals seems to be crucial.

## Methods

### Study setting and data collection

The study was conducted in a research and teaching hospital in a metropolitan area in the North part of Italy: Fondazione IRCCS Ca′ Granda – Ospedale Maggiore Policlinico, Milan. The Fondazione is a research and teaching hospital with three emergency units (adult, paediatric and obstetric), kidney, liver, lung, cornea and bone marrow transplant centres, a Medical School, several post-graduate schools and some 3 year courses for healthcare providers of the Faculty of Medicine and Surgery of the University of Milan. It also hosts a training centre for postgraduate courses and first and second level Master’s Degrees.

A new incident reporting database has been implemented in the hospital organization in 2014 [19]. The reporting of the fall event is recorded contextual to the event through an online template form. The incident reporting form collects the following general information: patient information (age, sex, type of hospital admission), clinical details (diagnosis, pharmacological therapy, fall risk assessment score) and fall event details (date, setting, consequences, causes, risk factors).

The fall risk assessment had to be performed through the STRATIFY tool at the time of hospitalization’s admission for patient older than 75 years in all units and for all patients in Neurology and Neuro-surgery units. Over the years, it has been recorded a higher number of fallers in Neurology and Neurosurgery units therefore it has been reinforced the fall prevention strategy accordingly, assessing all inpatients irrespectively of the age. Moreover, the fall risk assessment was strongly recommended for all inpatients showing recent fall history, dementia, hip fracture, diabetes type II, Parkinson’s diseases, arthritis, depression, functional limitations with need of aids, altered state of consciousness or cognitive disorders, visual and balance deficits, assumption of more than three drugs, incontinence, physical restraints, and osteoporosis.

The database was retrospectively searched for the events occurred between January 2014 and March 2015. All data in the database were anonymized so neither individual patient consent nor ethical approval were required.

Since we had access exclusively to data included in the web incident reporting database, only data of patients who experienced a fall, admitted from January 2014 to March 2015, were available. Thus, data were limited to this population. Falls occurring in all units of the hospital were included in data collection.

### Outcomes

Having collected all inpatients that experienced a fall, the primary outcome was to identify the proportion of true high-risk fallers (True Positive Rate, TPR), i.e. inpatients with STRATIFY score ≥ 2 that really experienced a fall, and the proportion of false low-risk fallers, i.e. inpatients identified as low-risk patient that experienced a fall howsoever (False Negative Rate, FNR), with their 95% confidence intervals. We also reported details of falls comprising extrinsic and intrinsic factors in all inpatients fallen and in the two subgroups, low and high risk, classified according to the STRATIFY.

### Data analysis

Data were presented descriptively with Chi-square significance testing for the comparison of extrinsic and intrinsic factors in the low/high risk subgroups and significance testing for the proportions TPR and FNR. For patients classified at high risk measures to prevent the fall are put in place; it is expected that the TPR should not be very high and it may not be considered as the sensitivity of the STRATIFY test which quantify the “a priori” proportion of true positive patients without intervention. The second proportion instead allows to quantify the false negative rate of STRATIFY in our organization and to give the size of the phenomenon which requires different evaluation criteria in order to have further reduction of falling risk. We generated an excel report with all data for each patients and an anonymous ID. Mean, median and frequencies described sample characteristics and fall events. Mean, median, interquartile range and standard deviation were used to synthetize the STRATIFY score and so the risk level of falling. The significant level for all the tests is 0.05.

## Results

We collected 399 incident reports received from 40 units. After removal of duplicates, 365 reports of fall were included in the analysis. The fall rate was 0.9 per 1000 patient-days in 2014.

### Characteristics of inpatients who fell

Among the 365 falls reported, 56.7% (*n* = 207) of fallers were men and 43.3% (*n* = 158) women. The median age was 72 (Interquartile Range 59–82). Women fallers median age was 71 (Interquartile Range 54.25–81) while men median age was 72 (Interquartile Range 59.5–82). Half of the fallers were hospitalized in an ordinary admission (49.6%, *n* = 181) while 45.5% (*n* = 166) were admitted from the emergency department. The major number of fallers was more likely to be admitted because of internal medicine conditions (35.6%, *n* = 130), followed by surgery (18.9%, *n* = 69), neurologic diseases (15.9%, *n* = 58), cardiologic problems (8.2%, *n* = 30), respiratory disorders (7.7%, *n* = 28), psychiatric conditions (3.6%, n = 13), pediatric problems (3.6%, n = 13), gynecologic conditions (2.5%, *n* = 9) and orthopedics (0.1%, *n* = 2).

### Fall risk assessment with the STRATIFY tool

**Fig. 1:**
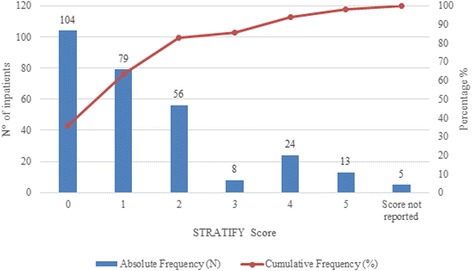
Fallers distribution per STRATIFY score

The fall risk assessment with the STRATIFY was performed in 289 (79.6%) out of 363 incident reports. For 2 out of 365 records was not possible to extrapolate whether the STRATIFY assessment was done or not due to lack of information, therefore a total number of 363 records was used for the analysis. Among the 289 records performed the STRATIFY assessment, 5 did not report the score. Overall, the mean score of STRATIFY asse**s**sment was 1.3 ± 1.4 and the median value was equal to 1. Figure 1 showed the proportion of subjects for each possible total score of the STRATIFY assessment on 284 cases. The cumulative frequency showed that the TPR, our primary outcome, was 35.6% (*n* = 101, 95% CI 30% - 41.1%) while the FNR, our secondary outcome, 64.4% (*n* = 183, 95% CI 58.9% - 70%).

Of the 74 out of 363 (20.4%) fallers for which the risk of falling was not evaluated, classifying those subjects straight as at low risk, the 72.4% (*n* = 55) should have been assessed instead since they have at least one criterion (i.e. age > 75, previous fall, urinary incontinence) for which the STRATIFY was strongly recommended by the internal organizational procedure of the hospital for fall prevention, as detailed above.

### Characteristics, intrinsic and extrinsic factors of fall

**Fig. 2:**
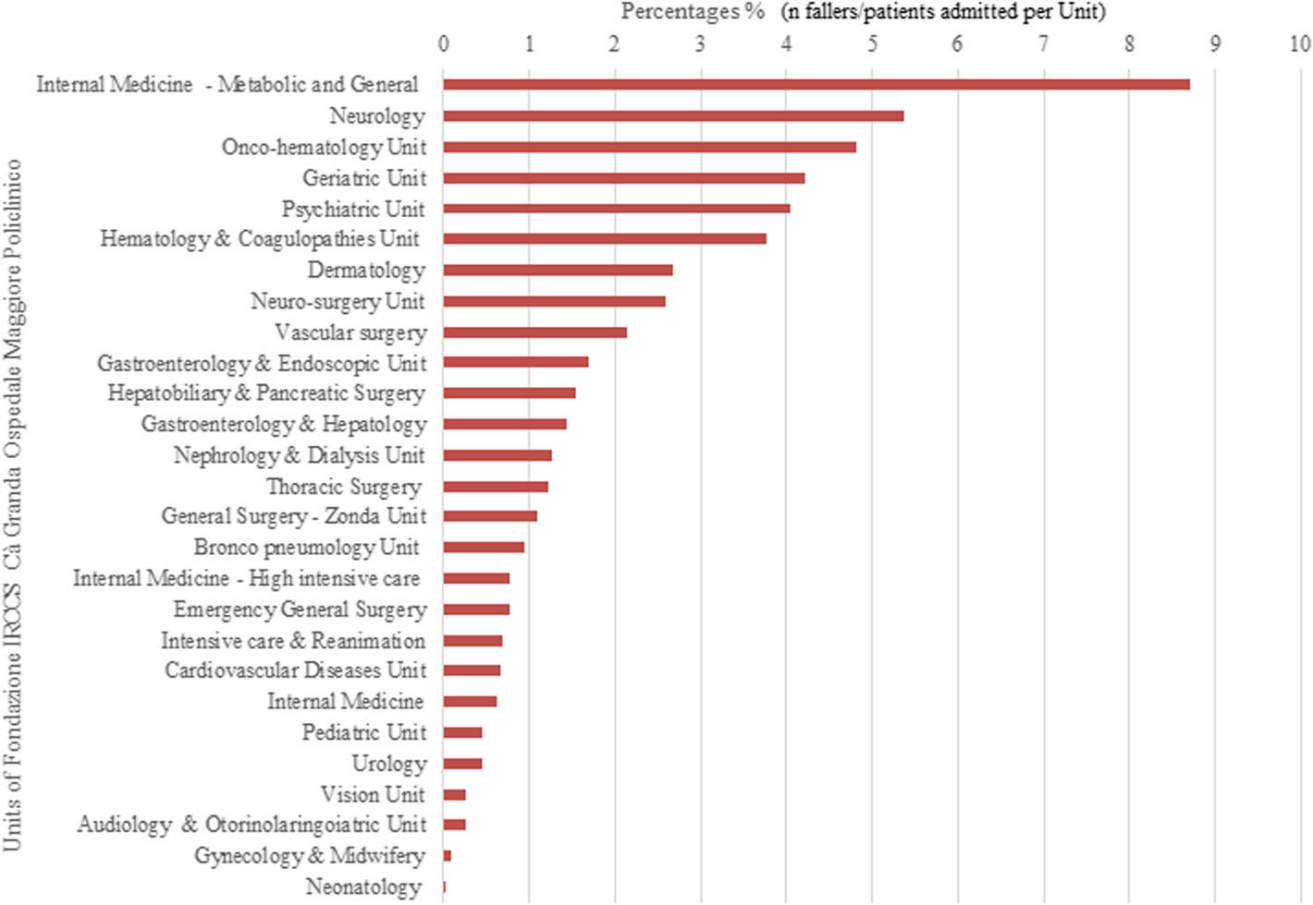
Fallers distribution on total inpatients admitted per each Unit

Among the 365 incident reports, 349 (95.6%) were real falls and 16 (4.4%) were classified as near falls (defined as an event that could have resulted in a fall, but did not). Figure 2 showed the distribution (%) of fallers on the total number of inpatients admitted in each Unit during the observed period. The top three Units with the highest frequency of falls among admitted inpatients were: Internal Medicine – Metabolic Unit (8.71%; 21/241), Neurology (5.37%; 44/819) and Onco-hematology Unit (4.82%; 21/436). While 12/365 fallers (3.3%) were outpatients admitted at the emergency room.

The majority of fall events happened in the patient room (63%, *n* = 230) followed by the toilette (22%, *n* = 82) and the corridor of the ward (7%, *n* = 26). During the working days (77.3%, *n* = 282) 45% of falls happen in the night, while during holiday days (23%, *n* = 83) falls occurred more during the morning shift (42.2%). Fall injuries were more of slight entity, less than 3 days of prognosis (38%, *n* = 113), severe entity was only in 9 events and no event occurred in death.

Tables 1 and 2 show the absolute and relative frequencies of intrinsic and extrinsic risk factors in fallers inpatients also in the two subgroups of low and high risk according to STRATIFY. The Chi-square test for the frequencies of intrinsic and extrinsic risk factors performed to compare the two subgroups of low/high risk patients are statistically significant (*p* < .05).

**Table 1:** Distribution of intrinsic risk factors in the total sample, in low risk and high risk patients’ subgroups

Intrinsic risk factors	Absolut and relative frequency in the 365 fallers n(%)	Absolut and relative frequency in 183 low risk patients n(%)	Absolut and relative frequency in 101 high risk patient n(%)
Cognitive impairment	130 (35.6)	66 (36.1)	45 (44.6)
Balance disorders	102 (28.0)	53 (29.0)	33 (32.7)
Neuromuscular and musculoskeletal disorders	100 (27.4)	49 (26.8)	37 (36.6)
Past falls	81 (22.2)	31 (16.9)	30 (29.7)
Incontinence	26 (7.1)	13 (7.1)	12 (11.9)
Visus impairment	25 (6.9)	14 (7.7)	9 (8.9)
Hearing impairment	15 (4.1)	6 (3.3)	6 (5.9)

**Table 2:** Distribution of extrinsic risk factors in the total sample, in low risk and high risk patient’s subgroups

Extrinsic risk factors	Absolut and relative frequency in the 365 fallers n(%)	Absolut and relative frequency in 183 low risk patients n(%)	Absolut and relative frequency in 101 high risk patient n(%)
Open footwear	73 (20)	26 (14.2)	33 (32.7)
Lack of lighting	58 (15.9)	35 (19.1)	19 (18.8)
Physical restraints	47 (12.9)	24 (13.1)	9 (8.9)
Distance from toilette	37 (10.1)	17 (9.3)	15 (14.9)
Walking aids (i.e. walker)	35 (9.6)	17 (9.3)	14 (13.9)
Inappropriate furniture	20 (5.5)	12 (6.6)	7 (6.9)
Wet floor	13 (3.6)	10(5.5)	2 (2.0)
Irregular floor	6 (1.6)	3 (1.6)	3 (3.0)

## Discussion

Reduction of inpatient risk of falling is an important health policy issue due to physical, psychological and socio-economic costs fall related. A frequent and attractive approach to prevent and reduce falls ‘number is to adopt and use a risk assessment tool. Despite the evidence [7, 16], a fall risk evaluation is performed only in 34% of the general elder population in contrast to the 70% of elderly receiving appropriate care for hypertension and heart failure, [20]. It has been highlighted how is problematic the transfer of the evidence supporting the effectiveness of fall prevention into clinical practice: a disparity between the wealth of the evidence and the neglect of falling exists, [21].

Overall, our fall rate was 0.9 per 1000 patient-days in 2014, a low rate in agreement with the range of published incidence rates of falls, [2, 9]. Collecting the number of falls per unit, we discovered that the absolute frequencies of falls on the total of the inpatients admitted per unit varied substantially across them, reflecting those that Laket et al. [22] found: the units of internal medicine are those with the highest rate of falls among inpatients admitted. The presence of patients suffered from co-morbidities and acute medical conditions may be the reason of the high prevalence in units as internal medicine besides external and environmental risks that contributes to increase the hazards, [23]. Nevertheless, the reported rate among units could be underestimated: in a previous study was underlined that operators may not consider the web-based incident reporting system as an instrument for improving health care quality and therefore, be not supportive in the use of risk management system, [19]. The difference falls number rate among units may be linked to the aversion of recording adverse events bound up with the health professionals, without pro-active “risk management” behavior. This attitude may reflect their wrong perception of low risk patients and, consequently, it might have been affected the likelihood of reporting a fall.

This study demonstrated that the risk assessment tool chosen by the organization, the STRATIFY, seems to be not adequate in detecting about 2/3 of fallers. The creator of the STRATIFY himself concluded that the elements of this tool (i.e. previous fall, agitation or stability) can vary between different inpatient populations (i.e. psychiatric, orthopedic, neurological), [24]. Oliver et al. suggested that case mix, ward design, type of patients, personnel skills may influence the validity and reliability of the risk assessment tool and so, a perspective validation is needed in any hospitals, [8, 11]. Indeed, according to several, the predictive accuracy and the external validity of the STRATIFY seems to be failed and not transferable to every hospital inpatient populations, [3, 25]. It should be also taken into consideration that this tool not assessed all the risks towards to patients are usually exposed. A non-appropriate screening tools leads to inappropriate fall prevention strategies and, consequently, to an unbalanced distribution of organizational resources in terms of educational programs, personnel and care assistance.

Our study revealed the inappropriateness of the use of STRATIFY as the only tool for fall prevention program implemented in our hospital as the Italian guideline required. The different distribution of intrinsic and extrinsic risk factors in the low/high risk subgroup, which generally are more in the second one, confirms the different causes for low risk patient fallers. However, the latter also suggests that possible other causes not already considered must be identified and monitored to prevent falls in low risk STRATIFY subgroup or to implement the use of other tool. For the high risk group the forbidden use of open footwear seems a simple intervention to prevent falls. Despite the definition of specific requirements for assessing inpatients at the admission, a percentage of fallers were not assessed for fall risk, i.e. STRATIFY was not performed, although they must have been assessed having risks factors as those recommend to be assessed. This can reflect a failure in the prevention program regarding its adherence by the health personnel. Even with the organization’ adherence to the national guidelines, [16], it seems to be a lack not only in the management system but also in the guideline recommendation. The most recent NICE guideline recommends to not limit the risk assessment at using fall predictive tools (i.e. STRATIFY or Morse assessment tool, [26]) and it suggests to consider a multidimensional fall assessment followed by a multifactorial intervention, [4]. Assessing all risk factors (patient-related and environmental) at early admission time should enable to correctly allocate the patient in low, moderate and high risk of falling, so that a cost-effective prevention program including physiotherapy and occupational therapy could be implemented. Gait and balance evaluation, daily living activities and home environment appraisal, along with assessing cognition and medication use, should be especially evaluated in those who are more inclined to be at high risk of falling. It should be remembered that patient ‘conditions can be altered during in-hospital stay, thus the assessment has to be a dynamic process.

In agreement with the NICE recommendations, a recent review suggest to organize the screening evaluation in three steps: (1) develop a fall management strategy and policy, (2) include a multifactorial assessment, and (3) allocate patients to interventions as physiotherapy or occupational therapy, [15]. In the viewpoint of a multifactorial assessment, the functional ability, gait and balance are key factors for developing an adequate preventive intervention. There are several tools used to assess them: the Time Up and Go Test (TUG), [27], the Short Physical Performance Battery, [28], the Tinetti Performance Oriented Mobility Assessment [29], the 6 Min Walking Test or the literature-based FRAT-up assessment, [30, 31]. Some of these tools are implemented in national guideline as the Best Practice Guidelines for Australian Residential Aged Care Facilities’ and in national preventive program as the STEADI program, developed by the Centre for Disease Control and Prevention in USA (https://www.safetyandquality.gov.au/our-work/falls-prevention/falls-prevention-resources, https://www.cdc.gov/steadi/index.html). On the contrary, Italian regional and national guidelines suggest to use predictive tool as the STRATIFY tool or the Morse scale being not specific to functional ability and equilibrium skills. The lack of updated preventive strategy including more “functional” evaluation maybe affect the success of the fall screening in our hospitals and so underestimate the burden of fall and its consequences.

Further research is needed to determine the optimal assessment and interventions for in-patients Italian population but also to implement a national standard program that fill the gap between recent evidence and still neglect in clinical practice. It is known that cost-effective programs would be more supportive if leadership is involved and powerful monitoring strategies are developed in order to ensure cooperation among staff from different disciplines [32]. Therefore, future studies should discover an easy, quick and multidimensional tool that may go beyond the limited resources in terms of time, personnel and money but always aim to the evidence based interventions.

### Limitation

Our observational study was a retrospective evaluation just of people who fell and its nature could be recognized as a limitation. We did not have the opportunity and the resources to select controls. Missing data could have been occurred because of the complexity of the management database system as well as the compliance of the several health professionals involved in managing it. It has been known that about 25% of fall events are not reported in incident reports but they are in medical charts [33], and so even our incident reporting system may be not effective as it is expected to be. A little sample of our medical charts obtained by the wards with the higher number of falls were reviewed to assess this and data obtained confirmed the published data.

## Conclusions

Evaluation of prevention programs should be considered as a feedback to improve the efficacy and the effectiveness of organization’s efforts in ensuring patient ‘safety. Our risk management program was found to be in line with the lower values of published incidence rates of falls but to be failure in adequately detecting all in-patient who actually experienced a fall. The multifactorial nature of falls requires the assessment of multiple domains including gait, balance, functional mobility, home/hospital environment and cognitive function. Multidimensional tool is needed in order to supply the STRATIFY’s limitations. Accordingly, it is essential to involve several healthcare professionals in developing the most adequate and appropriate risk assessment strategy: identifying incorrect needs of patients lead to allocate resources for erroneous priorities and untargeted interventions, decreasing healthcare performance and quality.

## Abbreviations

FNR: False Negative Rate
TPR: True Positive Rate
